# GLP-1RA precision medicine in people with type 2 diabetes: current insights and future prospects

**DOI:** 10.1172/JCI194742

**Published:** 2026-01-16

**Authors:** Pedro Cardoso, John M. Dennis, Ewan R. Pearson

**Affiliations:** 1 Clinical and Biomedical Sciences, Royal Devon & Exeter Hospital, University of Exeter Medical School, Exeter, United Kingdom.; 2 Division of Molecular & Clinical Medicine, Ninewells Hospital and Medical School, University of Dundee, Dundee, United Kingdom.

## Abstract

Glucagon-like peptide-1 receptor agonists (GLP-1RAs) have become an essential drug class for treating type 2 diabetes, offering proven benefits in glycemic control, weight reduction, and cardiovascular and renal protection. However, growing evidence of heterogeneity in GLP-1RA treatment effects highlights the potential for developing precision medicine approaches to more accurately allocate GLP-1RAs to maximize patient benefit. In this Review, we explore the evidence for treatment effect heterogeneity with GLP-1RAs, focusing on clinical and genetic factors that robustly influence established therapeutic outcomes. We also highlight the potential of recent predictive models that integrate routine clinical data with personalize treatment decisions, comparing GLP-1RA to other major type 2 diabetes drug classes. While such models have shown considerable promise in identifying optimal type 2 diabetes treatment based on glycemic response, their utility for informing treatment choice for other clinical outcomes remains largely unexplored.

## Introduction

Precision diabetes medicine is described in the recent second international consensus report as an approach to make more accurate clinical decisions, provide safer and more effective treatments, improve cost efficiency, and provide better access and adherence compared with conventional evidence-based medicine. The focus is on moving beyond standard treatment strategies toward tailoring diagnostics or treatment options for specific subgroups of populations that share meaningful clinical and biological characteristics (1). When considering precision treatment, a key requirement is the identification of robust treatment effect heterogeneity, recently defined in the Predictive Approaches to Treatment effect Heterogeneity (PATH) statement as “variation in the magnitude or direction of a treatment effect across levels of a covariate against a clinical outcome” (2). Once robust treatment effect heterogeneity is identified, the next step is to establish whether heterogeneity is sufficient to predict clinically meaningful differences in treatment benefits and harms at the individual or subgroup level, thus enabling more individualized prescribing. In type 2 diabetes (T2D), the ideal application of precision treatment would consider all available treatment options and predict which of these would be best for an individual or subgroup based on glycemia, weight, and longer-term outcomes such as cardiovascular or renal disease. In this Review, our focus is on the precision deployment of glucagon-like peptide-1 receptor agonists (GLP-1RAs), with a particular emphasis on clinical and genetic features that differentially alter the response to GLP-1RAs compared with other classes of T2D drugs.

GLP-1RAs were first introduced as a treatment for T2D in 2005 and have evolved over the past 20 years from twice-daily injections to once-weekly injections and daily oral tablets, as well as receiving marketing approval for the treatment of obesity. Here, we consider the GLP-1RA medications that have been widely available in clinical practice for the treatment of T2D, including semaglutide, dulaglutide, liraglutide, and exenatide, as well as the dual GLP-1R/glucose-dependent insulinotropic polypeptide receptor (GIPR) agonist tirzepatide. We do not aim to identify and review evidence on the treatment effect heterogeneity of GLP-1RAs specifically in people with obesity, and we consider only well-established T2D treatment outcomes, comprising short-term (glycemic response, weight reduction, and tolerability) and longer-term (cardiovascular and renal risk) endpoints. We first review the overall effects of GLP-1RA on these outcomes, before summarizing established and emerging evidence on clinical and genetic predictors of treatment effect heterogeneity. Finally, we provide an overview of recently published clinical prediction models for GLP-1RA treatment effect heterogeneity (alongside other available glucose-lowering therapies) and discuss their clinical implications for precision treatment in T2D. Notably, emerging evidence points to the potential for broader benefits of GLP-1RAs, such as managing the multiple facets of diabetic foot ulcers (3, 4), reduced lower-extremity amputation (5), and treatment of neurodegenerative disorders (6), but these are not discussed in depth here.

## GLP-1RA effectiveness in treating T2D

All GLP-1RAs achieve meaningful improvements in hemoglobin A_1c_ (HbA_1c_) and weight reduction in people with T2D, although effectiveness varies depending on the specific agent. For HbA_1c_ reduction, the most recent randomized control trial (RCT) network meta-analysis showed the largest effect with injectable semaglutide (1.40%, 95% CI 1.67% to 1.12%), followed by dulaglutide (1.09%, 95% CI 1.34% to 0.84%), liraglutide (1.04%, 95% CI 1.30% to 0.79%), and exenatide (0.81%, 95% CI 1.15% to 0.48%) (7). In particular, meaningful dose-response relationships for HbA_1c_ reduction were observed with injectable semaglutide and dulaglutide (7–9). In prior meta-analyses, oral semaglutide showed comparable efficacy to dulaglutide and liraglutide (8, 10).

In terms of weight reduction, injectable semaglutide (with dose-dependent effects) and liraglutide are more effective than placebo, whereas dulaglutide and exenatide have modest or negligible effects (7, 10). These weight reduction and dose-dependent findings are consistent across multiple meta-analyses, reinforcing the reliability of these comparisons (7, 8, 10, 11). Overall, while all GLP-1RAs offer clinical benefits, injectable semaglutide provides the greatest improvements in both glycemic control and weight loss, supporting its rapid adoption in managing T2D in contemporary clinical practice worldwide.

Beyond glycemic control and weight loss, GLP-1RAs may offer meaningful protection from long-term complications of T2D, including reduced risk of major adverse cardiovascular events (MACE). Meta-analyses indicate that GLP-1RA use is associated with a 13% reduction in MACE (pooled hazard ratio [HR] 0.87, 95% CI 0.81 to 0.93) (12), with broadly similar benefits observed across different GLP-1RA subtypes (8, 12, 13). Notably, however, while these effects are clinically meaningful, they are less pronounced in relative terms than the protection from heart failure seen with SGLT2 inhibitors (SGLT2i; HR 0.63, 95% CI 0.50 to 0.80) (14, 15), another T2D drug class with established benefits. Emerging data also suggest a potential benefit of GLP-1RAs for kidney outcomes, with a recent meta-analysis indicating a 19% lower risk of composite kidney events with GLP-1RAs (HR 0.81, 95% CI 0.72 to 0.92), although most data were derived from secondary analyses of cardiovascular outcome trials (12). HRs for macroalbuminuria, marked decline in estimated glomerular filtration rate (eGFR), kidney failure, and kidney-related mortality with GLP-1RAs have similarly ranged from 0.79 to 0.83 across multiple meta-analyses (12, 13, 16, 17). FLOW, the only kidney outcome trial to date of a GLP-1RA, included people with T2D and chronic kidney disease and found a 24% lower risk with injectable semaglutide (HR 0.76, 95% CI 0.66 to 0.88) (18). Again, these risk reductions are less pronounced than the well-established renal benefit of SGLT2i (meta-analysis HR 0.62, 95% CI 0.56 to 0.68) (19).

The GIPR/GLP-1R coagonist tirzepatide has demonstrated greater efficacy for both glycemia and weight than single agonists. A meta-analysis reported a mean HbA_1c_ reduction of 2.10% (95% CI 1.74% to 2.47%), with a meaningful dose-response relationship for HbA_1c_ (7). Similarly, tirzepatide achieved the highest levels of weight reduction compared with single GLP-1RAs. The potency of tirzepatide, especially for weight loss, was greater than expected from previous studies of dual agonism of GLP-1R and GIPR, as well as the relatively weak effect of GIPR agonism alone. The potential mechanisms underlying this enhanced efficacy are discussed elsewhere (7, 20–22). Regarding long-term outcomes, trial and observational data are limited; however, results from the SURPASS-CVOT trial (23) assessing the superiority of tirzepatide compared to dulaglutide for time to first occurrence of a MACE event were recently presented at the EASD 62nd annual meeting in 2026 (24), and one recent real-world study suggests use of tirzepatide may be associated with lower risks of MACE and adverse renal outcomes when compared with other GLP-1RA therapies (20).

A recent RCT meta-analysis has demonstrated that, over a median follow-up of 25 months, treatment discontinuations due to adverse events are more frequent with GLP-1RAs than placebo (12.7% vs. 9.2%; HR 1.51, 95% CI 1.18 to 1.94), although total serious adverse events are not increased (HR 0.95, 95% CI 0.90 to 1.01) (12). Among individual agents, injectable semaglutide (odds ratio [OR] 2.61, 95% CI 1.56 to 4.37), exenatide (OR 2.39, 95% CI 1.14 to 4.98), and liraglutide (OR 2.15, 95% CI 1.26 to 3.69) showed similarly increased rates of discontinuation, whereas the dulaglutide discontinuation rate (OR 1.41, 95% CI 0.86 to 2.29) may not differ significantly from placebo (7). The risk of tirzepatide discontinuation is also elevated compared with placebo (OR versus placebo 2.30, 95% CI 1.30 to 4.09) (7). Real-world evidence suggests a higher discontinuation rate of GLP-1RAs compared with that observed in trials, but the value varies widely between studies, ranging from 21.2% to 45.2%, with one study in the United Kingdom (UK) reporting a 31.2% discontinuation rate at one year after initiation (25–30). This variability is partly due to differing definitions and methods of capturing discontinuation. In addition, prescription gaps of 90 to 180 days are commonly used to infer discontinuation, but these may capture temporary interruptions rather than actual cessation in some cases (25, 31, 32).

## Heterogeneity of treatment effects for GLP-1RAs

**Figure 1:**
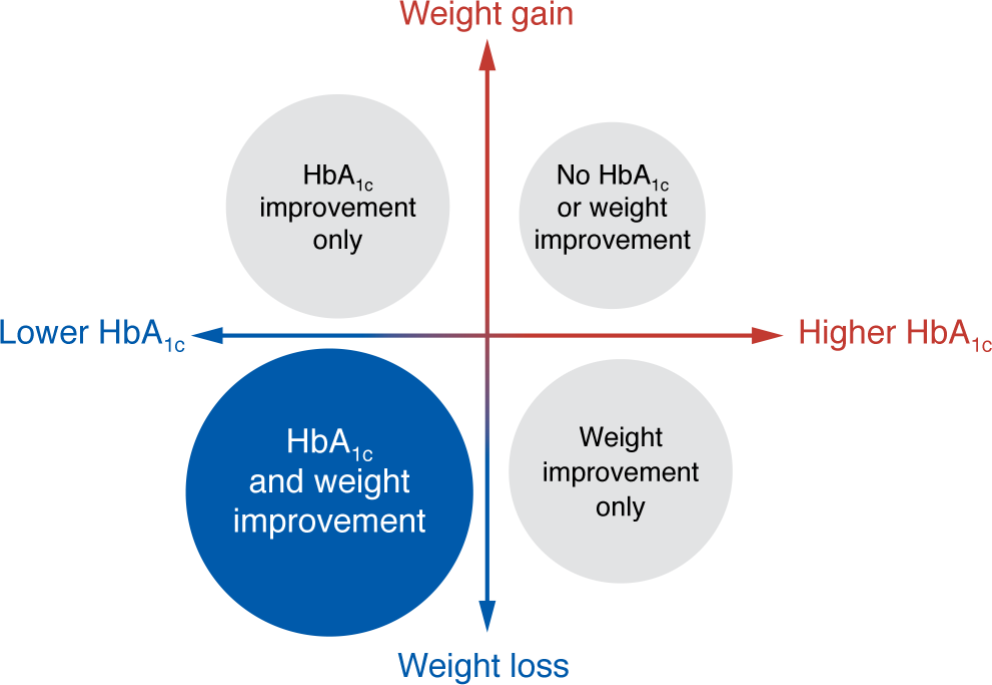
Individual-level variation in treatment response after initiating a GLP-1RA therapy. Most individuals initiating GLP-1RA therapy experience both glycemic and weight benefits. However, some achieve weight loss but minimal improvement in glycemic control, while others have a large reduction in HbA_1c_ with minimal or no weight loss.

As with all interventions, individual responses to GLP-1RAs vary. Most individuals initiating a GLP-1RA will experience both HbA_1c_ and weight benefits; however, some will achieve weight loss but minimal improvement in glycemic control, while others have a large reduction in HbA_1c_ with minimal or no weight loss (Figure 1). Although there is considerable variation in responses, this observation does not establish whether true treatment effect heterogeneity exists, that is, whether specific patient characteristics consistently influence outcomes and could thus support a precision medicine approach to GLP-1RA therapy. Differences in glycemic and weight changes may reflect factors such as adherence, persistence, and other influences beyond the underlying biological variability in drug response. To determine the presence of treatment effect heterogeneity, specific study designs and meta-analysis methods are needed. Below, we discuss studies that provide insight into the heterogeneity of GLP-1RA treatment effect.

## Short-term outcomes: HbA_1c_, weight, and tolerability

For a recent systematic review of treatment effect heterogeneity of GLP-1RAs, refer to Young et al. (15).

**Figure 2:**
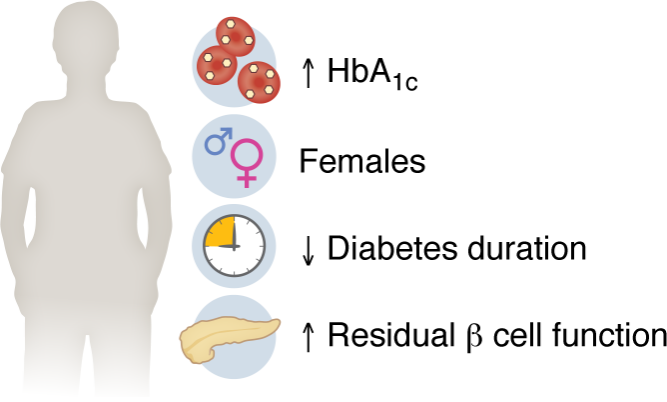
Determinants of increased HbA1c response on GLP-1RA treatment. Clinical and demographic characteristics are associated with increased short-term treatment response to GLP-1RAs.

Multiple studies have demonstrated that clinical and demographic characteristics are associated with differences in short-term treatment responses to GLP-1RAs (Figure 2), highlighting the potential to target GLP-1RA therapies to patients with specific characteristics (15). Similar to all T2D glucose-lowering therapies, a higher baseline HbA_1c_ is associated with greater glycemic response to GLP-1RAs (9, 15). Alongside that, greater weight reductions are observed in individuals with higher BMI, although interestingly, trial data have demonstrated less weight loss in individuals with higher baseline HbA_1c_ levels, potentially related to gastrointestinal motility problems observed in patients with diabetes (11, 33, 34).

There is also increasing evidence of clinically meaningful differences in GLP-1RA outcomes by sex, with women showing both more weight loss (9, 11, 35) and a greater glycemic response (36, 37) than men. The difference in glycemic response was recently replicated in post hoc analyses of the Harmony clinical trial and the PRIBA study (36, 38), although other observational studies have shown no clear sex differences (15, 39). A potential explanation for an increased glycemic response is that women have higher circulating GLP-1RA concentrations at equivalent doses (9). For a recent review of sex-specific differences and potential mechanisms, see Rentzeperi et al. (40).

Impaired β cell function has also been shown to be associated with a lower glycemic response to GLP-1RA. The PRIBA study prospectively assessed GLP-1RA treatment outcomes in 620 participants with T2D and found that a lower glycemic response was associated with markers of impaired β cell function, including longer diabetes duration, lower fasting C-peptide levels, and severe insulin deficiency (41). This aligns with the known mechanism of action of GLP-1RA as a potentiator of β cell insulin secretion (42), and data from other RCTs and observational studies have shown that a shorter duration of diabetes is associated with an improved glycemic response (15, 38, 43). Data on treatment effect heterogeneity by race or ethnicity are limited, although one RCT meta-analysis showed evidence of a higher HbA_1c_ response to GLP-1RAs in individuals of Asian versus non-Asian ethnicity, potentially related to the former’s lower baseline BMI (44). Finally, recent data suggest that features of metabolic syndrome, including hypertension, fatty liver, and obstructive sleep apnea, may be independently associated with a reduced weight loss response to GLP-1RAs (45).

To date, most evidence about factors influencing the risk of GLP-1RA treatment discontinuation comes from observational rather than clinical trial data. In a recent UK observational study, where universal health care minimizes the confounding effect of financial influences on prescribing, older age, longer diabetes duration, and history of prior metformin discontinuation were each associated with a higher risk of discontinuing GLP-1RAs within 12 months (25). Women were more likely to discontinue treatment at 3 and 6 months, but by 12 months, discontinuation rates were similar between men and women. Higher discontinuation rates were observed among people of South Asian and Black ethnicities compared with those of White ethnicity. Additionally, patients with a lower BMI (<30 kg/m^2^) and lower eGFR (<75 mL/min/1.73 m^2^) were more likely to discontinue a GLP-1RA therapy. Lim et al. (31) also reported an association between lower BMI and higher discontinuation rate, although further studies are needed to determine whether these differences simply reflect existing diabetes and obesity management guidelines, which means GLP-1RAs are mainly prescribed to those with a BMI greater than 30 kg/m^2^. This highlights a major challenge when interpreting differences in discontinuation from real-world datasets; as such, studies of large and unselected populations do not capture the underlying reasons for discontinuation.

Clinical predictors for the short-term efficacy of tirzepatide appear to be broadly similar to those identified for the single GLP-1RAs. Post hoc analyses from the SURPASS 1–4 trials series identified female sex, White or Asian ethnicity, younger age, lower HbA_1c_ and lower non-HDL cholesterol as predictors of greater weight loss (33, 46, 47). The association between higher HbA_1c_ and reduced weight loss has been reported elsewhere (48, 49), although the underlying mechanisms are not fully understood. Potential explanatory factors include attenuation of treatment efficacy by background therapies, impaired appetite regulation and, by extension, dietary adherence, potentially altered microbiome, and genetic predisposition to weight gain (50). Regarding glycemic outcomes with tirzepatide, post hoc analysis from RCT data has suggested younger people with a shorter duration of diabetes, lower HbA_1c_, and better β cell function were more likely to achieve an HbA_1c_ of 58 mmol/mol (46, 51).

## Longer-term cardiovascular and renal outcomes

Post hoc analyses of cardiovascular and renal outcome trials, as well as meta-analyses, have so far relied primarily on subgroup analyses and have provided only limited insights into potential GLP-1RA treatment effect heterogeneity (52–54). RCT subgroup analyses suggest that the relative cardiovascular and kidney benefits of GLP-1RAs persist in lower-risk groups without established cardiorenal disease (55). A recent meta-analysis showed that the relative reduction in MACE with GLP-1RA use was lower for older compared with younger participants (HR per 30-year increase in age 1.47, 95% CI 1.07 to 2.02), in contrast to SGLT2i, for which greater benefits with increasing age were observed (HR 0.76, 95% CI 0.62 to 0.93) (56). Another meta-analysis suggested that while GLP-1RA cardiorenal benefits are evident in White, Asian, and other populations, they may be limited for Black participants (57). Aside from these findings, meta-analyses have not shown other statistically significant heterogeneity in cardiovascular outcomes across subgroups defined by baseline HbA_1c_, sex, BMI/obesity, chronic kidney disease, diabetes duration, background glucose-lowering therapy, history of microvascular disease, or GLP-1RA dosing interval (12, 15). Regarding renal outcomes, post hoc analyses indicate greater eGFR preservation in patients with lower BMI and reduced baseline kidney function, although results varied between trials (12, 15).

Real-world studies including broad and diverse populations of people with T2D may offer potential insights into GLP-1RA effects beyond those gathered from clinical trials. However, to date, few studies have been conducted to specifically evaluate treatment effect heterogeneity on cardiovascular and kidney outcomes (15). One study by Raparelli et al. identified a lower risk of cardiovascular events with GLP-1RAs versus sulfonylureas in women (HR 0.57, 95% CI 0.48 to 0.68), but this difference was not present in men (HR 0.82, 95% CI 0.71 to 0.95) (58). That study involved 167,254 American adults with T2D uncontrolled with metformin, a lower-risk group than the majority of participants included in GLP-1RA outcome trials (58). An understudied area is the importance of patient-level heterogeneity in absolute risk of cardiovascular and kidney outcomes alongside the relative benefits of GLP-1RAs. Although real-world evidence suggests GLP-1RAs maintain their relative benefit for primary prevention in people without preexisting cardiovascular and renal disease, absolute therapeutic benefits will be smaller in those individuals due to lower baseline risk (55, 59). Conversely, those at a higher baseline risk will gain the greatest absolute benefit from GLP-1RAs, even with similar relative effects. This highlights that consistent relative treatment effects naturally lead to heterogeneous absolute risk reductions, and emphasizes the potential clinical relevance of accurate baseline risk stratification as an approach for optimizing GLP-1RA prescribing. Recent research focused on SGLT2i therapy for kidney disease prevention in people with T2D has shown that using baseline risk, rather than a treatment-guideline-informed threshold of albuminuria of 3 mg/mmol or higher, better identifies those likely to benefit most from SGLT2i therapy initiation (60), and the same approach could be applied in future studies to evaluate GLP-1RAs.

## Genetic predictors of treatment effect heterogeneity

Several studies have investigated the role of genetic variation in the heterogeneity of response to GLP-1RA treatment. However, many of those studies are underpowered, lack replication, and primarily examine variants in *CTRB1/2* (61) and *GLP1R* (62, 63). Here, we focus on three more robust studies with larger sample sizes and/or replication.

Dawed et al. used both clinical trial and observational data, employing a candidate gene approach (*GLP1R*), a common variant genome-wide association study (GWAS), and a rare variant burden test (64). The clinical trial data included 1,771 individuals treated with albiglutide from the Harmony trials and 1,562 individuals treated with dulaglutide from the AWARD phase III trials, while the observational data comprised 1,238 individuals treated with exenatide and liraglutide. Two candidate variants in the *GLP1R* gene were assessed, with the Gly168Ser variant showing a small but significant effect on glycemic response (0.08 mmol/mol worse response per serine). For the GWAS, no genome-wide significant variants were identified. However, the rare variant burden test identified variants in *ARRB1* to be associated with a greater glycemic response to GLP-1RAs, with carriers of one or more rare alleles having a 2.2 mmol/mol greater HbA_1c_ reduction compared with individuals with the reference allele. The signal was primarily driven by the p.Thr370Met variant, which is rare in White Europeans (0.05%) but present in 6% of Hispanics and 11% of American Indians, suggesting a greater genetic contribution of *ARRB1* to GLP-1RA treatment heterogeneity in these populations. Notably, *ARRB1* variants were not associated with weight reduction, indicating a pancreas-specific role for these variants. *ARRB1* encodes arrestin-β1, which is involved in recycling G protein–coupled receptors to the cell surface. One putative mechanism for the variants in *ARRB1* that increase the efficacy of GLP-1RAs in lowering HbA_1c_ could be increased expression of the GLP-1R at the β cell membrane.

A second paper, currently in preprint, reports that diabetes-associated variants in the gene encoding peptidyl-glycine α-amidating monooxygenase (*PAM*) are linked to GLP-1 resistance in vivo and a reduced glucose-lowering effect with GLP-1RAs (65). In a meta-analysis of three observational studies (*n* = 1,119), noncarriers of loss-of-function alleles in *PAM* experienced a 13.6 mmol/mol reduction in HbA_1c_ with liraglutide and exenatide treatment. This effect was reduced by 6.0 mmol/mol in p.S539W carriers (*P* = 0.025) and by 2.7 mmol/mol (*P* = 0.05) in p.D563G carriers. There was no significant effect of *PAM* variants on response to albiglutide (Harmony phase III trials), suggesting that the impact of *PAM* variants may vary depending on the specific GLP-1RA used.

Finally, a recent large biobank-based study examined the role of BMI and T2D polygenic risk scores, as well as variants in the *GLP1R* or *PCSK1* gene in GLP-1RA–associated weight loss (66). Although not the focus of this Review, the study’s robust methodology warrants discussion. Researchers used nine population biobanks to identify 6,750 individuals, both with and without diabetes, who had initiated a GLP-1RA therapy, with a weight measurement in the 12 months before and 6–12 months after initiation. Consistent with other studies, higher baseline BMI and female sex were associated with greater absolute weight loss from GLP-1RA treatment. However, neither polygenic risk scores for T2D or BMI, nor variants in the *GLP1R* or *PCSK1* gene were associated with GLP-1RA–induced weight loss (66). Therefore, unlike the glycemic response to GLP-1RAs, no genetic variants have yet been robustly linked to weight loss outcomes.

When evaluating the overall contribution of genetics and its clinical implications, the study by Dawed et al. (64) sets its findings into context. That study found that 4% of the population with low-frequency variants in *ARRB1* responded 30% better (an absolute benefit of 3.2 mmol/mol) to GLP-1RAs compared with the 9% of the people with the reference *ARRB1* allele but two *GLP1R* variants. Since the average yearly glycemic deterioration is 1 mmol/mol (67), the authors suggest that these 4% of individuals could experience an additional 3 years before treatment failure, potentially supporting earlier use of GLP-1RAs in this subgroup. Implementing such an approach would require genotyping at the time of prescribing and a clinical trial to evaluate genotype-guided treatment before widespread adoption in clinical care. However, given current HbA_1c_ reductions achieved by modern GLP-1RAs and dual agonists, there is presently insufficient evidence to support precision medicine using genetics for GLP-1RA therapy.

Does this mean that genetic variation does not contribute to GLP-1RA response? No studies have reported on the heritability of glycemic or weight response to GLP-1RAs, but studies in twins report a 53% heritability for GLP-1–induced insulin secretion (68), and SNP-based heritability estimates indicate a degree of heritability for other diabetes medications (34% for metformin and 37% for sulphonylureas; ref. 69). Therefore, it is reasonable to assume that GLP-1RA response is a moderately heritable trait. Genetic studies on drug response are generally underpowered, except for common variants, but sequencing studies have identified several functional coding variants in *GLP1R* that may influence GLP-1RA treatment response. For example, a sequencing study of 8,642 Danish individuals found 36 nonsynonymous variants in *GLP1R*, 10 of which were predicted to cause loss of function. Notably, carriers of loss-of-function variants showed only minor increases in glucose levels, with no meaningful differences in cardiometabolic traits (70). Conversely, a UK Biobank (https://www.ukbiobank.ac.uk/) study identified 60 variants across four signaling pathways (cell surface expression, complete or pathway-specific gain of function, and loss of function). Variants that reduced GLP-1R cell surface expression (but not other functional variants) led to higher HbA_1c_ and BMI (71). As sequencing initiatives expand in large biobanks, such as the UK Biobank and All of Us (https://www.researchallofus.org/), and as access to genetic data from clinical trials grows, we will soon be better equipped to investigate how rare variants that alter GLP-1 function or cell surface expression affect GLP-1RA efficacy in individuals with and without diabetes.

## Precision medicine models for GLP-1RA treatment effect heterogeneity

The development of prediction models for treatment effect heterogeneity is an area of growing interest in diabetes research and across clinical medicine (2, 66). Predictive approaches aim to combine multiple features into algorithms that predict treatment effect heterogeneity at the subgroup or individual patient level. These algorithms typically predict outcome differences between two or more treatment options, providing direct information to guide clinical decisions regarding optimal treatment based on the underlying predictive features in the algorithm. By integrating multiple features, predictive approaches can address some limitations of traditional “one-at-a-time” subgroup analyses, which are often underpowered in clinical trials and tend to have low reproducibility (72).

A novel Bayesian machine learning–based model has recently been developed to predict treatment effect heterogeneity, specifically the differential 6-month glycemic response to GLP-1RAs versus SGLT2i, using routine clinical features (36). In 27,319 individuals from UK primary care, the model identified 20.3% as having a clinically relevant predicted HbA_1c_ benefit exceeding 3 mmol/mol with GLP-1RAs over SGLT2i. Key predictors of greater glycemic benefit with GLP-1RAs included female sex, lower baseline HbA_1c_, better kidney function (as measured by eGFR), and older age. In contrast, BMI, other routine biomarkers, diabetes complications, and common comorbidities did not meaningfully alter the prediction of the optimal therapy. The strong performance of the model on hold-back UK data demonstrates the clear potential of model-based approaches to target these therapies using routine features, although further validation in independent cohorts is a necessary next step.

**Figure 3:**
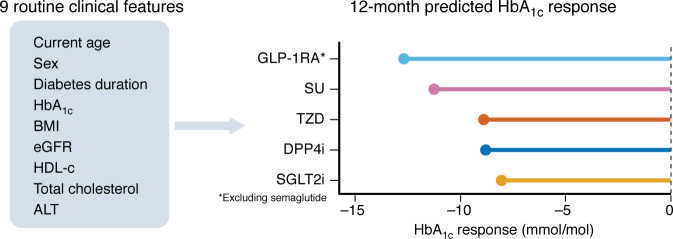
Schematic of the five-drug class model published in Dennis et al. (38). The model was developed to produce individualized predictions of 12-month HbA_1c_ treatment response to five major drug classes using routine clinical features. Prototype calculator available at https://www.diabetesgenes.org/t2-treatment/ GLP-1RA, glucagon-like peptide-1 receptor agonists; SU, sulfonylureas; TZD, thiazolidinediones; DPP4i, dipeptidyl peptidase-4 inhibitors; SGLT2i, sodium–glucose cotransporter-2 inhibitors.

This precision medicine approach for optimizing glycemic response was extended in Dennis et al. (38), leveraging routine clinical features to predict the 12-month HbA_1c_ response to five major glucose-lowering medication classes following metformin: GLP-1RAs, SGLT2i, DPP4 inhibitors (DPP4i), thiazolidinediones, and sulfonylureas (Figure 3). The model demonstrated potential to optimize treatment selection and improve clinical outcomes, with the predicted optimal therapy leading to an average 12-month HbA_1c_ benefit of 5 mmol/mol, a 38% lower risk of 5-year glycemic failure, and a 14%–29% reduction in 5-year risk of diabetes complications. As an important validation step, calibration of predicted differences in 12-month response was tested in a reanalysis of clinical trial data. For the UK cohort used in the study (212,166 drug initiations), GLP-1RAs were predicted to be the optimal glucose-lowering therapy for 33.4% of the population, while the other therapies were evenly split (aside from DPP4i, <0.01%). However, a meaningful amount of variation is hidden in this overall measure, as stratifying by sex revealed that GLP-1RAs were identified as the optimal glucose-lowering therapy for 71.9% of drug initiations in women but only 9.3% of initiations in men. The lesser relative benefit of GLP-1RAs among those with longer diabetes duration was also evident, with GLP-1RAs predicted as the optimal therapy for 40.7% of those with shorter diabetes duration (3 years), compared with only 27.8% among those with longer duration (10 years). An important area for future work will be to incorporate semaglutide and tirzepatide as newer agents into the algorithm, considering their increasing clinical use and superior efficacy compared with earlier GLP-1RAs. In parallel, assessing the added value of genetic predictive markers beyond routine clinical features could further refine precision (73). Crucially, such developments will require rigorous validation in independent external cohorts, as reliance solely on internal validation risks overfitting and limits generalizability. The model’s sole focus on optimizing glycemia is also a limitation for clinical deployment, and future algorithms that take into account other outcomes, including weight loss, side-effects, and reduction in cardiovascular and renal risk, are needed.

In contrast to these promising results for glycemic response, we are aware of only one study to date that has assessed the potential for predicting heterogeneity in risk of short-term GLP-1RA discontinuation. Considering the five major glucose-lowering T2D medication classes, including GLP-1RAs (excluding semaglutide and tirzepatide), the recently proposed model, developed using UK observational data, showed limited predictive utility (area under the receiver operating characteristic curve [AUROC] = 0.61) for predicting discontinuation within 3 months of initiation (25). Although this study suggests limited utility for targeting GLP-1RAs at the individual patient level based on broad discontinuation risk, it does not preclude the possibility of developing more specific models to predict GLP-1RA–specific side effects to inform targeted therapeutic decisions. Given the relatively small sample size and restricted inclusion criteria of T2D clinical drug trials, the application of causal methods in representative large-scale real-world patient populations receiving GLP-1RAs may be necessary to generate robust evidence for heterogeneity in side-effect risks (74).

## Future directions and conclusions

In this Review, we highlighted the emerging contemporary evidence on heterogeneous treatment effects for GLP-1RAs and GIPR/GLP-1R dual agonists, identifying clinically relevant effect modification for glycemic and weight outcomes, but a lack of evidence of effect modification for cardiovascular and kidney outcomes. In particular, recent data suggest that routinely measured clinical features, such as sex, diabetes duration, and residual β cell function, are associated with differences in glycemic response, while greater weight reductions are observed in individuals with higher baseline BMI and less weight loss in those with higher baseline HbA_1c_ levels. Although the effects of individual clinical and genetic features are generally modest, recent model-based approaches further support the notion that combining clinical features to estimate heterogeneity in glycemic response for individual patients has potential as a precision medicine approach to inform the selection of optimal therapy when choosing between GLP-1RAs and other T2D treatment options after metformin. While genetic variants also influence the response to GLP-1RA treatment, their contribution to overall heterogeneity is currently modest. Together, these insights demonstrate a clear potential for a precision medicine approach to optimize the use of GLP-1RAs in clinical care.

A key next step toward achieving a comprehensive precision medicine approach for GLP-1RAs will be to systematically assess treatment effect heterogeneity across a broader range of outcomes, including cardiovascular, kidney, and mortality endpoints, side effects, and patient-reported outcomes, ideally with direct comparison to other T2D therapies. Evaluation of treatment effect heterogeneity for longer-term outcomes will likely require the sharing of clinical trial data to support individual participant data meta-analysis, as well as the design of specifically tailored large-scale observational studies to fill evidence gaps due to the restricted sample size, inclusion criteria, and lack of active comparator arms in GLP-1RA outcome trials. RCTs are, however, uniquely positioned to robustly capture side-effect profiles, while observational data and patient-reported outcome measures can complement trial data by shedding light on real-world tolerability and the impacts on quality of life.

Second, a greater understanding of the influences of nonroutine biomarkers, genetics, and other omics on treatment effect heterogeneity will be essential. Rapid advances in genetics, particularly the development of large-scale biobanks and the sequencing of clinical trial cohorts, are likely to lead to more powerful polygenic risk scores and the discovery of rare variants that alter GLP-1RA outcomes. Beyond genetics, the utility of molecular biomarkers, including metabolomics and proteomics, for precision GLP-1RA medicine has yet to be established. Similarly, the utility of recently proposed T2D subtypes remains to be determined, although their potential to improve T2D treatment selection is likely to be limited (75, 76). Importantly, these efforts must prioritize including multiple-ancestry cohorts to ensure the generalizability and equity of emerging tools.

Finally, the therapeutic landscape is rapidly expanding. We have moved rapidly from a limited number of GLP-1RAs to a rapidly expanding and diverse range of incretin-based agents, now including dual and triple agonists, as well as oral formulations. Although robust clinical data on interindividual variability with these newer agents remain limited, it is crucial that future precision medicine frameworks anticipate their integration.

Extending existing work on treatment effect heterogeneity with GLP-1RAs in this way to encompass enhanced predictive biomarkers, the newest GLP-1–associated agents, and broader clinical outcomes may offer the potential for a comprehensive precision medicine approach to GLP-1RA therapy to optimize treatment and meaningfully improve the clinical care of people with T2D.

## Version 1 01/16/2026

Electronic publication
